# The Impact of Digital Inequities on Esophageal Cancer Disparities in the US

**DOI:** 10.3390/cancers15235522

**Published:** 2023-11-22

**Authors:** David J. Fei-Zhang, Evan R. Edwards, Shravan Asthana, Daniel C. Chelius, Anthony M. Sheyn, Jeffrey C. Rastatter

**Affiliations:** 1 Northwestern University Feinberg School of Medicine, 420 E Superior St, Chicago, IL 60611, USA; evan.edwards@northwestern.edu (E.R.E.);; 2 Department of Otolaryngology—Head and Neck Surgery, University of Tennessee Health Science Center, 910 Madison Avenue, Memphis, TN 38163, USA; 3 Department of Otolaryngology—Head and Neck Surgery, Baylor College of Medicine, 6701 Fannin Street, Houston, TX 77030, USA; 4 Department of Otolaryngology—Head and Neck Surgery, Northwestern University Feinberg School of Medicine, 675 N Saint Clair, Chicago, IL 60611, USA; jrastatter@luriechildrens.org

**Keywords:** social determinants of health, internet access, broadband service, technology infrastructure, esophageal cancer, esophageal cancer

## Abstract

**Simple Summary:**

This study aims to understand how unequal access to digital technology in the United States affects the care and outcomes of people with esophageal cancer. The researchers developed a Digital Inequity Index to measure this inequality in different areas. They found that as digital inequality increases, esophageal cancer patients have shorter follow-up times and lower survival rates. They are also less likely to receive important surgeries and chemotherapy. These findings show that unequal access to the internet and technology can significantly impact the care that cancer patients receive. This research emphasizes the need to address these disparities and provide equal access to technology for better healthcare outcomes in the future.

**Abstract:**

Background: There is currently no comprehensive tool that quantifiably measures validated factors of modern technology access in the US for digital inequity impact on esophageal cancer care (EC). Objective: To assess the influence of digital inequities on esophageal cancer disparities while accounting for traditional social determinants. Methods: 15,656 EC patients from 2013–2017 in SEER were assessed for significant regression trends in long-term follow-up, survival, prognosis, and treatment with increasing overall digital inequity, as measured by the Digital Inequity Index (DII). The DII was calculated based on 17 census tract-level variables derived from the American Community Survey and Federal Communications Commission. Variables were categorized as infrastructure access or sociodemographic, ranked, and then averaged into a composite score. Results: With increasing overall digital inequity, significant decreases in the length of long-term follow-up (*p* < 0.001) and survival (*p* < 0.001) for EC patients were observed. EC patients showed decreased odds of receiving indicated surgical resection (OR 0.97, 95% CI 0.95–99) with increasing digital inequity. They also showed increased odds of advanced preliminary staging (OR 1.02, 95% CI 1.00–1.05) and decreased odds of receiving indicated chemotherapy (OR 0.97;95% CI 0.95–99). Conclusions: Digital inequities meaningfully contribute to detrimental trends in EC patient care in the US, allowing discourse for targeted means of alleviating disparities while contextualizing national, sociodemographic trends of the impact of online access on informed care.

## 1. Introduction

As esophageal cancers (EC) remain the seventh most prevalent cancer and sixth leading cause of cancer-related death globally [1,2,3], social determinants of health (SdoH) have long contributed to EC disparities in care and prognosis, namely related to low socioeconomic status, minority race–ethnicity, and increased rurality [3,4,5]. These studies and others have highlighted how SdoH comprise 80–90% of modifiable contributors to public health outcomes across EC and other diseases [6], relaying an urgent need to address such factors in improving EC burdens.

One potential strategy for tackling this EC burden is the use of the internet for disseminating knowledge to patients, providers, and the wider general audience. Within the broader area of gastrointestinal cancers, several investigations have observed how the usage and quality of online information elicits significant benefits to their diagnosis, treatment, prevention, and prognosis [7,8,9]. Though this positive impact has been thoroughly investigated, measures of how patients gain digital accessibility to these resources remain seldom inquired among those diagnosed with gastrointestinal cancers, let alone esophageal cancers specifically. This is in contrast to there being several national investigations of associating digital resource inequities with community health disparities in diabetes and obesity prevalence [10,11]. In turn, this “digital inequity”, which encompasses a community’s possession of internet-capable electronic devices, local network infrastructure, or economic broadband service pricing relative to the local income level, involves modern-day SDoH factors that are sparsely assessed in comparison to the research on traditional SDoH such as socioeconomic status, race–ethnicity, education, and others on EC disparities. 

Presently, there are few initiatives working to characterize this “digital inequity” within health disparities. State-level efforts, such as the Digital Divide Index (DDI) from the Rural Indiana Stats database, and national-level ones, such as the Federal Communications Commission (FCC) Connect2Health Broadband Map, have been developed for such purposes but fall short in multiple ways. Namely, the FCC Connect2Health Broadband Map is not updated regularly and the DDI has a very limited geographic scope [12,13]. With the validated set of sociodemographic and digital inequity variables established in these tools, alongside their publicly available data sources of the American Community Survey/US Census surveys and FCC National Broadband Reports, the expansion of these measures into a more contemporary, national scope presents an opportunity to observe larger digital inequity associations with national care and prognostic disparities.

**Figure 1:**
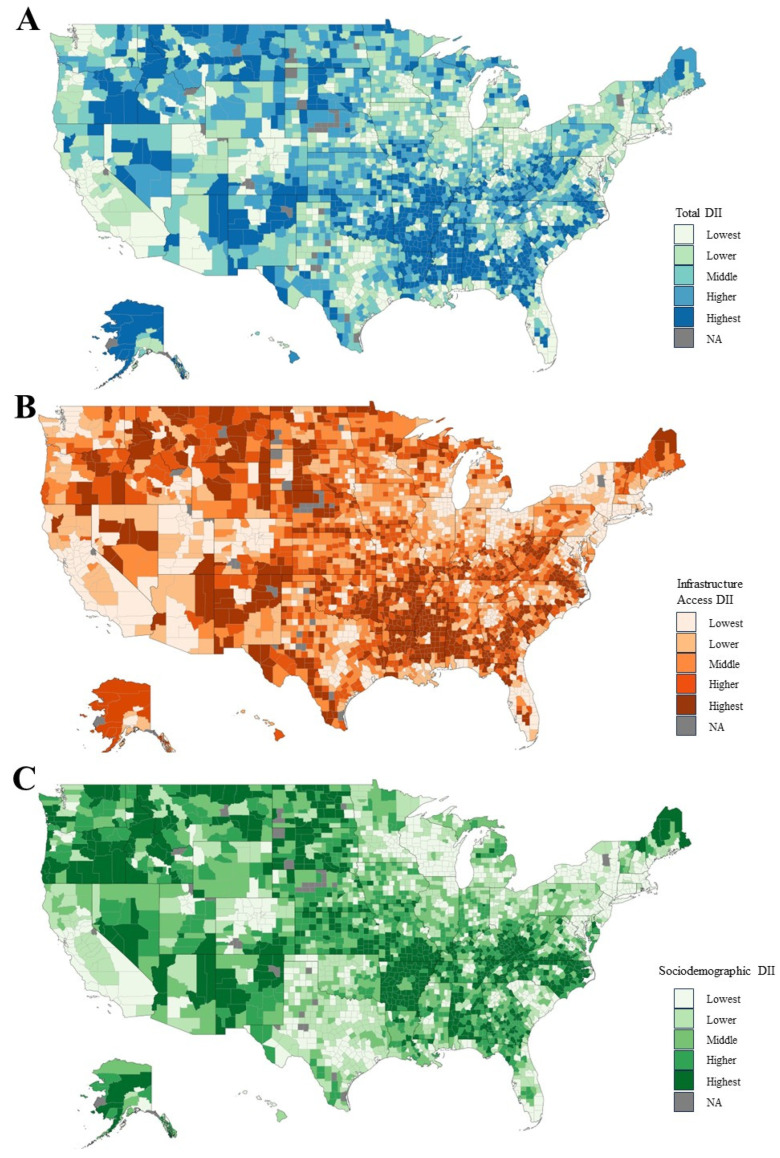
Distribution of total DII ranked scores across the US. Ranked digital inequity scores were assigned per county in the (**A**) total composite DII, (**B**) infrastructure access and usage, and (**C**) sociodemographic categories.

Using this validated multivariable approach, the Digital Inequity Index (DII) (Figure 1) was developed as a US-based, geographically differentiated tool to comprehensively assess a variety of broadband infrastructure, electronic device access, and internet access affordability while adjusting for traditional social determinants of health. Given the paucity of investigations into modern-day understandings of digital inequity impact on EC care and prognosis, we sought to apply the DII to quantifiably assess digital resource access across the country for their effects of “digital inequity” on EC care and prognosis. This study hypothesizes that national disparities of poorer clinical outcomes and decreased indicated treatment would occur with increasing levels of digital inequity while adjusting for traditional social determinants of health, such as socioeconomic status and disability status.

## 2. Materials and Methods

This retrospective cohort study follows the Strengthening the Reporting of Observational Studies in Epidemiology (STROBE) reporting guideline. No prior IRB/ethics committee approval or waiver of informed consent was needed; the databases queried consist of publicly available, de-identified data.

### 2.1. Data Sources

The DII was calculated based on 17 census tract-level variables derived from the 2018 American Community Survey (ACS) 5-year estimates from 2013–2017 and the Federal Communications 14th Broadband Report. Variables were extracted and grouped into two DII subcategories of “infrastructure access”—comprised of the measures representing households “without a desktop or laptop”, “without access to non-mobile broadband”, “without access to broadband: DSL”, “without access to broadband: cable”, “without access to broadband: fiber”, “without access to broadband: terrestrial fixed wireless”, “without a mobile or non-mobile internet subscription of any type”, “without an internet subscription of cable, fiber, or DSL”, “without a broadband subscription in households making $20,000 or less”, “without a broadband subscription in households making $20,000—$74,999”, “without a broadband subscription in households making $75,000 or more”—and “sociodemographic”—comprised of “25+-aged people without high school diploma”, “25+-aged people without an associate’s degree or higher”, “25+-aged people without a bachelor’s degree or higher”, “25+-aged people below the poverty level within the last 12 months”, “25+-aged people below 150% of the poverty level within the last 12 months”, and “25+-aged people with a disability status pertaining to cognitive, ambulatory, or self-care difficulties”. For reference, these are tabulated in Supplementary Table S1 as DII subcategories.

Ranked scores were then assigned to each ACS variable based on their relative value compared to all other census tracts nationwide. These were then adjusted by tract population to calculate weighted mean scores on the county level within their respective DII subcategories. The total composite DII score was calculated based on the combined means of the two subcategories to account for non-digital, sociodemographic confounders. DII scores were then arranged into five ordered classes by natural break (Jenks) classification by comparing the sum of the squared deviations between classes to each array mean and utilizing a goodness of variance fit. These five classes were then labeled as “Lowest”, “Lower”, “Middle”, “Higher”, and “Highest”, where “Highest” is considered the highest measured digital inequity (Figure 1). These variates and weighted calculations were validated in prior digital index tools from the Rural Indiana Stats and the FCC Connect2Health [12,13].

The National Cancer Institute—Surveillance, Epidemiology, and End Results Program (NCI-SEER) database contains national datasets of patient variables, pathological characteristics, treatment modalities, and prognostic outcomes. Months under surveillance represents a length-of-care measurement reflecting the active follow-up a patient receives for their primary malignancy up until the last provider interaction. Months-long survival represents active follow-up until the patient suffers a mortal outcome. Staging is based on SEER-designated variables labeled as “Stage IV”, “distant [expansion]”, or “distal [expansion]” and recoded under American Joint Committee on Cancer, Sixth Edition (AJCC-6) classifications. Primary surgery occurrence represents whether patients received surgery for their primary malignancy. 

DII scores were abstracted and matched to SEER patient data based on the county of residence at the time of diagnosis. The schematic workflow is provided in Supplementary Figure S1.

### 2.2. Population Definitions

SEER was queried for adult (20+ years) patients diagnosed with EC from 2013 to 2017. Primary sites were extracted using the International Classification of Diseases for Oncology, Third Edition (ICD-O-3) topographic codes [C150.0–C159.0].

### 2.3. Statistical Methods

Demographics tables were grouped by total DII scores delineated by the natural break (Jenks) classifications of “Lowest”, “Lower”, “Middle”, “Higher”, and “Highest”. 

The follow-up time/surveillance period and survival period were analyzed by total DII scores and DII subcategory scores. DII scores were split into relative, equivalently sampled quintiles based on actual DII scores. The relative DII quintiles were delineated by “<20”, “20–39.99”, “40–59.99”, “60–79.99”, and “80–99.99.” Among these total and DII theme quintiles, differences between the mean months under surveillance and the survival period for the lowest and highest DII-scored quintiles were calculated. Trend significance was assessed by linear regression across relative DII quintiles for both continuous measures, and box plots were generated to assess the median interquartile range (IQR), and 1.5 times the IQR. Mean values were also calculated per the relative quintile group. Survival months were analyzed similarly to months under surveillance. However, after separating patients into relative DII quintiles, patients who were alive/lost upon the last follow-up were excluded to extract patients who were dead upon the last follow-up. 

Logistic regression was used to assess the outcomes of primary surgery occurrence, advanced staging at the time of diagnosis (reference, “early staging” or “Stage I–III”; comparator, “late staging”, or “Stage IV”), and the receipt of radiation therapy or chemotherapy across DII quintiles (reference, lowest DII quintile; comparators, ordinally increasing levels of DII quintiles). A two-sided *p*-value of <0.05 was set as the threshold of statistical significance. All statistical analyses were conducted in R version 4.2.1.

## 3. Results

A total of 15,656 adult patients with primary EC were extracted from SEER. Of those, 65–84 years of age (*n* = 8268, 52.8%), male sex (*n* = 12,142, 77.6%), white race (*n* = 11,923, 76.2%), and those from Western regions of the US (*n* = 7397, 47.2%) were the most represented among the study population. Adenocarcinomas (*n* = 9445, 60.3%) and squamous cells (*n* = 4917, 31.4%) were the most represented histology subtypes. Further demographic and clinical characteristics stratified by total DII classes are noted in Table 1.

### 3.1. Trends in Months under Surveillance and Survival by Relative DII Percentile

**Figure 2:**
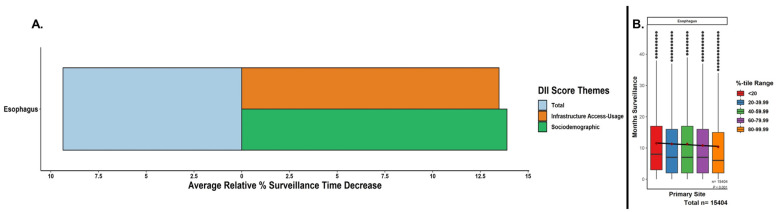
Relative decreases in months surveyed with increasing DII scores. (**A**) Percentage decreases from lowest to highest DII quintiles based on mean months surveyed for total DII score and subcomponent DII theme subscores. EC patients were assigned DII scores and split into relative quintiles. (**B**) A linear regression across all the represented values (i.e., not the mean values) in each of the boxplot quintiles was performed to assess for continuous trend significance of the surveillance period for increasing the total DII. Boxplots = median, IQR, 1.5*IQR; mean months surveyed per quintile = maroon diamonds; outliers = black dots; *p*-value for regression.

The mean time under surveillance stratified by total DII score quintiles is summarized in Figure 2. Overall, the mean surveillance time was significantly decreased (*p* < 0.001) when assessing the continuous regression trends from the lowest DII quintile (i.e., with the least digital inequity) to the highest DII quintiles (i.e., increasing digital inequity), after adjusting for non-digital, sociodemographic confounders. The mean differences between the lowest and highest quintiles were from 11.52 months to 10.44 months for a relative reduction of 9.36%. Contributing to this total DII trend, increasing inequity in the subcategories of infrastructure access and usage and sociodemographic factors showed an equivalent impact (Figure 2).

**Figure 3:**
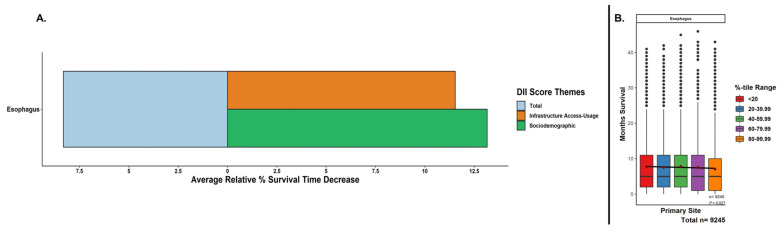
Relative decreases in months survival with increasing DII scores. (**A**) Percentage decreases from lowest to highest DII quintiles based on mean months survived for total DII score and subcomponent DII theme subscores. EC patients were assigned DII scores and split into relative quintiles. (**B**) A linear regression across all the represented values (i.e., not the mean values) in each of the boxplot quintiles was performed to assess for continuous trend significance of the survival period for increasing the total DII. Boxplots = median, IQR, 1.5*IQR; mean months survived per quintile = maroon diamonds; outliers = black dots; *p*-value for regression.

The mean survival period stratified by total DII score quintiles is summarized in Figure 3. Similar to the previous section, the mean survival time was significantly decreased (*p* < 0.001) when assessing continuous regression trends with increasing DII quintiles (i.e., lowest to highest). The mean differences between the lowest and highest quintiles were from 7.71 to 7.07 months for a relative reduction of 8.32%. Contributing to this total DII trend, increasing inequity within the subcategories showed higher contributions of sociodemographic factors compared to infrastructure access and usage factors (Figure 3).

### 3.2. Trends in Staging and Treatment

Logistic regression results for EC staging and treatment variables across total DII score quintiles are summarized in Table 2. Compared to the lowest quintile, EC was at increased odds of presentation at an advanced stage (OR 1.02; 95% CI 1.01–1.05) with significant contributions from both infrastructure access and usage and sociodemographic variable subcategories. The odds of receiving radiation therapy did not significantly differ for EC patients between the highest and lowest quintiles; however, EC patients were at decreased odds of receiving chemotherapy (OR 0.97; 95% CI 0.95–1.00). In terms of surgical resection, EC patients in the highest quintile demonstrated decreased odds of receiving indicated surgical intervention (OR 0.97; 95% CI 0.95–99).

## 4. Discussion

To our knowledge, this is the first and largest study to develop and implement a national index as a comprehensive measure for evaluating digital inequity while accounting for sociodemographic confounders across EC patients and evaluating the relationship between digital inequity in EC care and prognosis while accounting for non-digital, sociodemographic SDoH. Overall, increased digital inequity measured by total DII and its subcategories showed significant decreases in surveillance and survival periods, as well as increased odds of late disease staging and decreased odds of surgery receipt for EC patients.

Given the wide range of DII scores within our study population, the impact of digital inequity and its overlap with sociodemographic factors on EC disparities is of universal importance. Large-scale analyses of technology usage and internet access impact have become more crucial to understanding their health impact on EC and other cancer patients, especially within more rural areas with higher digital inequity. During the late 2000s and early 2010s, investigations were underway in underserved regions, such as Appalachia and Kentucky, which highlighted the increased cancer burden being connected to lower broadband access [14,15,16]. When digital infrastructure was implemented in these areas, such as added telephone and internet video conferencing for patients and clinic systems, both patients and providers reported higher satisfaction with cancer symptom management and higher acceptance, usage, and satisfaction of connected health technologies [16]. In turn, our study reaffirms these prior results and showcases unique methodologies for evaluating how such digital deficits can be targeted on a national scale.

Given the complex nature of the impact of SdoH on EC and other cancers, our Digital Inequity Index demonstrates the need for developing comprehensive SdoH tools that leverage modern large datasets to assess the interrelated, real-world impacts of SdoH. Prior efforts in tackling this issue for SdoH cancer studies have come in the form of social determinant indices, such as the Social Vulnerability Index and Area Deprivation Index [17,18,19,20,21,22,23,24,25,26,27,28,29], to quantifiably characterize the impact of traditional SdoH, such as socioeconomic status, minority race–ethnicity, and rurality–urbanicity, with real-world valence. Despite these indices being utilized to identify areas of higher social disadvantage and target prospective interventions for reducing cancer disparities, they lack the breadth of SdoH needed to assess key, modern-day factors of digital inequity that affect population health as significantly as traditional SdoH. Our findings of infrastructure access and usage inequity contributing equivalently to EC disparities while accounting for non-digital, sociodemographic factors support this assertion. Moreover, this supposition can be more directly observed within other global–regional studies, which have shown significantly fewer postoperative complications related to nutrition and quality of life with internet-based dietary management compared to without for EC patients [30]. Thus, our study highlights the need for the novel development of indices accounting for lesser-studied determinant factors, such as those encompassed by our DII. Ultimately, they allow nuanced, quantifiable characterizations of their impact while contextualizing the influence of both digital and traditional SdoH on EC health inequities.

The principal strengths of our study are that it utilizes a novel, comprehensive index to assess a wide variety of digital inequity determinants precisely measured by the American Community Survey and FCC broadband reports while accounting for non-digital/sociodemographic variables. It encompasses a large, contemporary population of EC patients across the US and looks into patient variables as well as level-of-care measurements and prognostic outcomes.

However, this study has limitations. The DII and SEER data only overlap from 2013 to 2017, which necessitates future infrastructure/census and patient data that should be more up-to-date. Given the majority white representation of our study populace, future studies revolving around race–ethnicity-based stratified groups should be performed to assess whether the impact of observed DII differences would change. The range of clinical characteristics from the standalone SEER database does not contain the full breadth of variables that would further characterize our findings, which would urge the use of other paid, SEER–Medicare-linked databases to provide additional information on operative details and treatment modalities.

## 5. Conclusions

Using the Digital Inequity Index, this study provides unique quantitative and qualitative contemporary digital SdoH-based assessments of the care and prognosis of esophageal cancer patients across the US. Our results not only reaffirm prior knowledge of past digital resource disparity studies but also expand upon them by incorporating a nationwide patient populace and adjusting for the complex interactions between digital and non-digital SdoH factors. Furthermore, they provide a means of identifying which and, more importantly, how much digital resource inequity contributes to overall disparity trends in the context of varied SdoH measures. Ultimately, our use of DII establishes a basis for future inquiry into SdoH-related esophageal cancer studies and for advising providers on which SdoH should be investigated to relay the most benefit, informing both practice and public policy toward the equitable delivery of esophageal cancer care.

## Figures and Tables

**Table 1:** Patient characteristics by DII score.

		Total Digital Inequity Index Category				
Characteristic	*n*	Lowest Total DII, *n* = 11,032 (70%)	Lower Total DII, *n* = 2328 (15%)	Middle Total DII, *n* = 1195 (7.6%)	Higher Total DII, *n* = 558 (3.6%)	Highest Total DII, *n* = 543 (3.5%)
**Age**	15,656					
20–44 Years		255 (2.3%)	42 (1.8%)	27 (2.3%)	15 (2.7%)	10 (1.8%)
45–64 Years		3842 (35%)	870 (37%)	477 (40%)	254 (46%)	242 (45%)
65–84 Years		5882 (53%)	1237 (53%)	624 (52%)	255 (46%)	270 (50%)
85+ Years		1053 (9.5%)	179 (7.7%)	67 (5.6%)	34 (6.1%)	21 (3.9%)
**Sex**	15,656					
Male		8481 (77%)	1790 (77%)	959 (80%)	465 (83%)	447 (82%)
Female		2551 (23%)	538 (23%)	236 (20%)	93 (17%)	96 (18%)
**Race**	15,656					
White		8300 (75%)	1834 (79%)	959 (80%)	434 (78%)	396 (73%)
Black		867 (7.9%)	324 (14%)	161 (13%)	87 (16%)	121 (22%)
Hispanic		1047 (9.5%)	121 (5.2%)	54 (4.5%)	10 (1.8%)	18 (3.3%)
Asian or Pacific Islander		709 (6.4%)	34 (1.5%)	11 (0.9%)	23 (4.1%)	0 (0%)
Native American		59 (0.5%)	11 (0.5%)	7 (0.6%)	1 (0.2%)	5 (0.9%)
Unknown		50 (0.5%)	4 (0.2%)	3 (0.3%)	3 (0.5%)	3 (0.6%)
**Region**	15,656					
Midwest		823 (7.5%)	700 (30%)	231 (19%)	56 (10%)	6 (1.1%)
Northeast		2018 (18%)	531 (23%)	85 (7.1%)	0 (0%)	0 (0%)
South		1669 (15%)	573 (25%)	647 (54%)	440 (79%)	480 (88%)
West		6522 (59%)	524 (23%)	232 (19%)	62 (11%)	57 (10%)
**ICD-O-3 Histopathology**	15,656					
Adenocarcinomas		6696 (61%)	1392 (60%)	734 (61%)	333 (60%)	290 (53%)
Squamous Cell Neoplasms		3455 (31%)	708 (30%)	374 (31%)	178 (32%)	202 (37%)
Epithelial Neoplasms, NOS		442 (4.0%)	110 (4.7%)	35 (2.9%)	22 (3.9%)	25 (4.6%)
Unspecified Neoplasms		338 (3.1%)	92 (4.0%)	44 (3.7%)	22 (3.9%)	23 (4.2%)
Complex Epithelial Neoplasms		101 (0.9%)	26 (1.1%)	8 (0.7%)	3 (0.5%)	3 (0.6%)
**TNM Combined Staging**	13,818					
Stage I–III		5678 (58%)	1186 (58%)	595 (56%)	254 (53%)	283 (60%)
Stage IV & Above		4089 (42%)	859 (42%)	466 (44%)	221 (47%)	187 (40%)
**No. of Primary Tumors by Dx**	15,060					
1		8144 (77%)	1746 (78%)	898 (78%)	446 (82%)	414 (79%)
2 or More		2446 (23%)	503 (22%)	253 (22%)	97 (18%)	113 (21%)
**Primary Surgery Performed**	15,027					
No Surgery		8114 (76%)	1676 (75%)	884 (78%)	404 (79%)	409 (81%)
Surgery		2537 (24%)	547 (25%)	252 (22%)	107 (21%)	97 (19%)
**Radiation Therapy Performed**	15,656					
No Therapy		5073 (46%)	1068 (46%)	555 (46%)	247 (44%)	251 (46%)
Therapy		5959 (54%)	1260 (54%)	640 (54%)	311 (56%)	292 (54%)
**Chemotherapy Performed**	15,656					
No Therapy		4349 (39%)	969 (42%)	501 (42%)	228 (41%)	223 (41%)
Therapy		6683 (61%)	1359 (58%)	694 (58%)	330 (59%)	320 (59%)
**Vital Status on Last Follow-up**	15,656					
Alive		4478 (41%)	875 (38%)	449 (38%)	185 (33%)	172 (32%)
Dead		6554 (59%)	1453 (62%)	746 (62%)	373 (67%)	371 (68%)

**Table 2:** DII-based analyses of staging and treatment receipt.

Outcome	DII Characteristic	OR	95% CI	*p*-Value
Advanced Staging	Total	1.02	1.00, 1.05	0.042
	Infrastructure Access & Usage	1.04	1.01, 1.06	0.003
	Sociodemographic	1.06	1.03, 1.08	0.000
Chemotherapy	Total	0.97	0.95, 0.99	0.028
	Infrastructure Access & Usage	0.96	0.94, 0.99	0.001
	Sociodemographic	0.97	0.95, 1.00	0.023
Radiation	Total	0.98	0.96, 1.00	0.105
	Infrastructure Access & Usage	0.98	0.95, 1.00	0.038
	Sociodemographic	1.00	0.98, 1.03	0.733
Surgical Resection	Total	0.97	0.95, 0.99	0.048
	Infrastructure Access & Usage	0.95	0.93, 0.98	0.000
	Sociodemographic	0.96	0.94, 0.99	0.008

Univariate logistic regressions across DII quintiles based on first presentation occurrence of Stage IV/distant expansion and primary treatment (surgery, radiation, chemotherapy) occurrence for increasing total DII score and subcomponent DII theme subscores per disease class.

## Data Availability

Due to designated data-sharing agreements with the Surveillance, Epidemiology, and End Results (SEER) administrators, we are unable to provide the linked datasets of this study readily.

## Supplementary Materials

The following supporting information can be downloaded at: https://www.mdpi.com/article/10.3390/cancers15235522/s1, Figure S1: Schematic workflow of DII and SEER database manipulation; Table S1: Variables used for DII development.
