# Supplementary material for: The Impact of Digital Inequities on Esophageal Cancer Disparities in the US
Source: Cancers (Basel). 2023 Nov 22;15(23):5522. doi: 10.3390/cancers15235522 (PMC10705088; doi:10.3390/cancers15235522)
Supplement: Supplementary file 1 [file cancers-15-05522-s001.zip › cancers-2635495-supplementary.pdf]

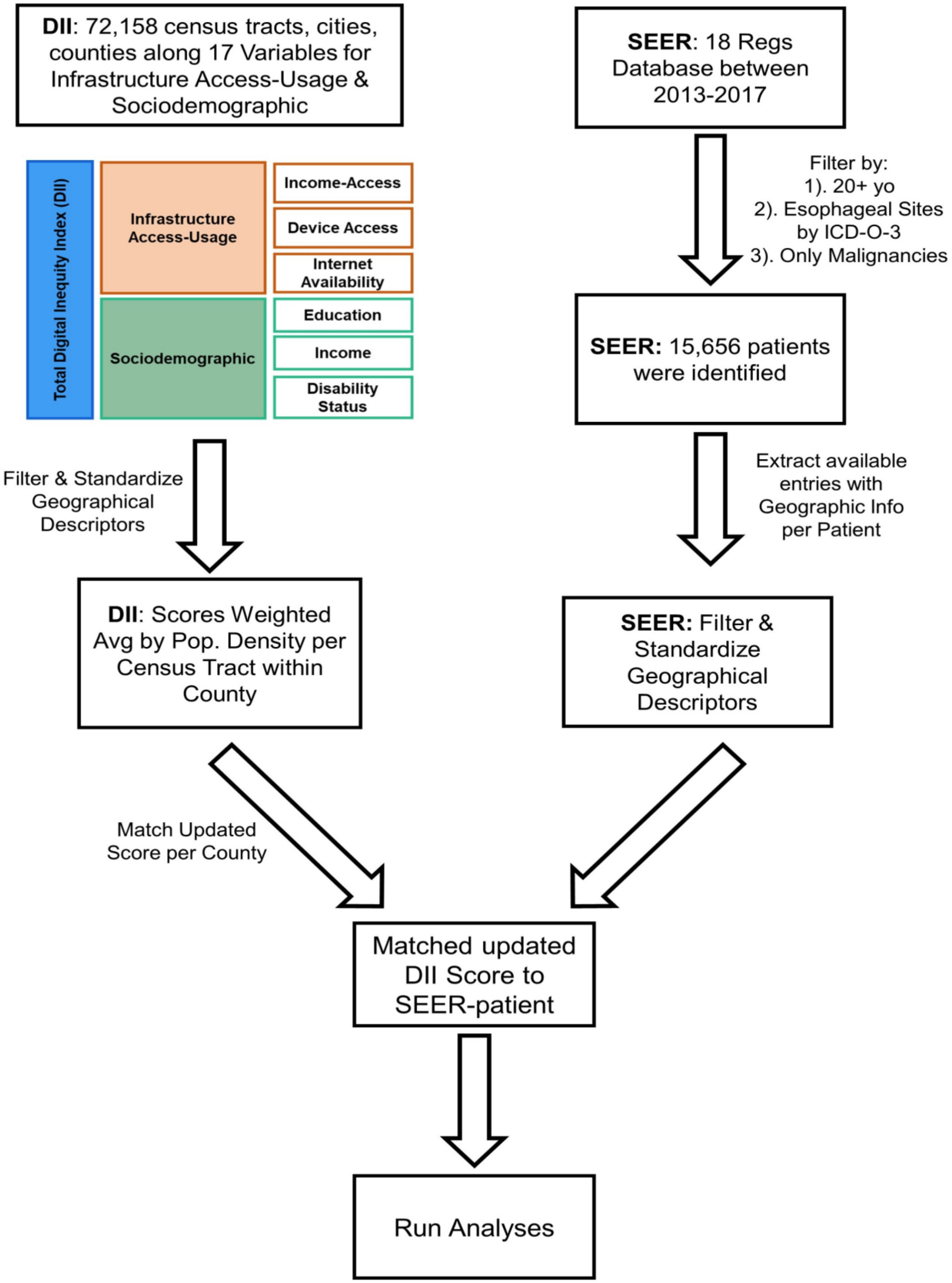

Supplemental Figure S1. Schematic Workflow of DII & SEER Database Manipulation. The Digital Inequity Index was merged with patient data from the SEER database through linked geocodes of County.

Supplement Table S1. Variables used for DII-development

Infrastructure-Access	Sociodemographic
Households without a desktop or laptop	25+ years-aged people without a high-school diploma
Without access to non-mobile broadband	25+ years-aged people without an associate’s degree or higher
Without access to Broadband - DSL	25+ years-aged people without a bachelor’s degree or higher
Without access to Broadband - Cable	Below poverty level within the last 12 months
Without access to Broadband - Fiber	Below 150% of the poverty level within the last 12 months
Without access to Broadband - Terrestrial Fixed WiFi	Disability status pertaining to cognitive, ambulatory, or self-care difficulties
Without mobile or non-mobile internet subscription of any type	
Without an internet subscription of cable, fiber, or DSL	
Without a broadband subscription in households $20,000 or less	
Without a broadband subscription in households making $20,000 - $74,999	
Without a broadband subscription in households making $75,000 or more	
